# Supplementary material for: The role of extracellular vesicle fusion with target cells in triggering systemic inflammation
Source: Nat Commun. 2024 Feb 7;15:1150. doi: 10.1038/s41467-024-45125-1 (PMC10850166; doi:10.1038/s41467-024-45125-1)
Supplement: Supplementary file 1 — Supplementary Information [file 41467_2024_45125_MOESM1_ESM.pdf]

The Role of Extracellular Vesicle Fusion with Target Cells in Triggering Systemic Inflammation

Praveen Papareddy[1], Ines Tapken[1,2,3], Keshia Kroh[1,4], Ravi Kiran Varma Bhongir[1], Milladur Rahman[5], Maria Baumgarten[1], Eda Irem Cim[1], Lilla Györffy[1], Emanuel Smeds[1], Ariane Neumann[1], Srinivas Veerla[6], Jon Olinder[7], Henrik Thorlacus[5], Cecilia Ryden[7], Eva Bartakova[8], Michal Holub[8], and Heiko Herwald[1]

[1]Division of Infection Medicine, Department of Clinical Sciences, Lund, Lund University, Sweden
[2]SMATHERIA gGmbH – Non-Profit Biomedical Research Institute, Hannover, Germany
[3]Center for Systems Neuroscience (ZSN), Hannover, Germany
[4]Department of Viroscience, Erasmus Medical Center, Rotterdam, the Netherlands
[5]Section of Surgery, Department of Clinical Sciences, Malmö, Lund University, Sweden
[6]Division of Oncology and Pathology, Lund, Department of Clinical Sciences, Lund University, Lund, Sweden
[7]Division of Infection Medicine, Helsingborg Hospital and Department of Clinical Sciences Helsingborg, Lund University, Sweden
[8]Department of Infectious Diseases, First Faculty of Medicine, Charles University and Military University Hospital Prague, Czech Republic

Running Title:

Systematic action of extracellular vesicles -mediated cell stimulation

Corresponding authors:

Heiko Herwald, Division of Infection Medicine, Department of Clinical Sciences, Lund, Lund University, Biomedical Center, Tornavägen 10, SE-22184 Lund, Sweden.
Email: heiko.hewald@med.lu.se
Phone: +46-46-2224182

Praveen Papareddy, Division of Infection Medicine, Department of Clinical Sciences, Lund, Lund University, Biomedical Center, Tornavägen 10, SE-22184 Lund, Sweden.
Email: praveen.papareddy@med.lu.se
Phone: +46-46-2226808

Supplemental Fig. 1

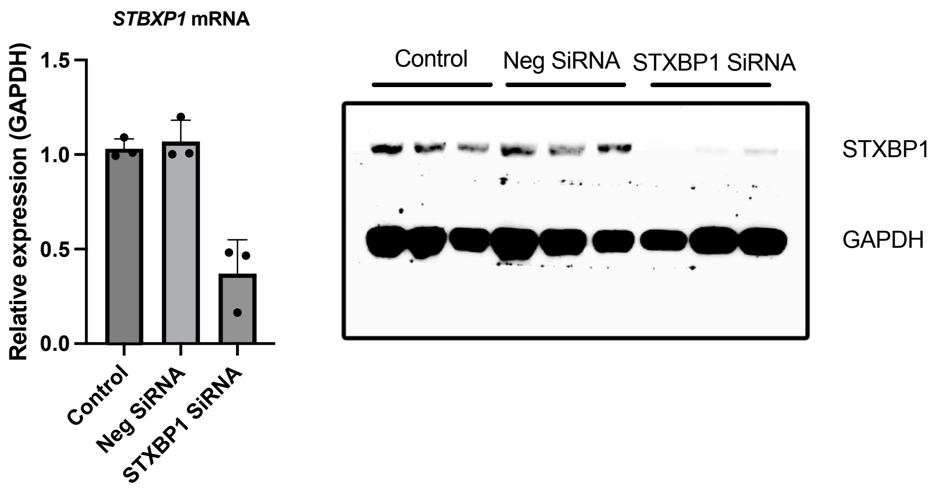

Supplementary Figure 1: Expression of *Stxbp1* in SiRNA treatment mice. P-values determined by ordinary one-way ANOVA with Tukey's multiple comparisons test (SEM; *n=3, mice per group*).

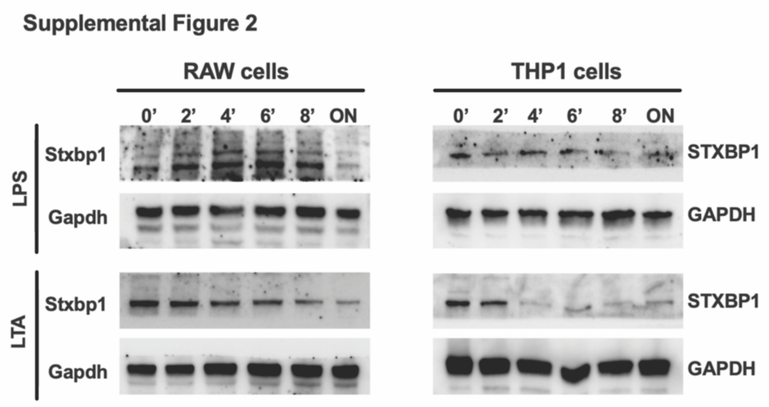

Supplementary Figure 2: Raw macrophages and THP1 were stimulated with LPS and LTA. Following the stimulation, Stxbp1 expression levels were determined at 0 h, 2 h, 4 h, 6 h, 8 h and overnight, with 0 h as a negative control. Stxbp1 expression levels are compared to GAPDH levels. Data represent a representative experiment from two independent experiments.

Supplemental figure 3

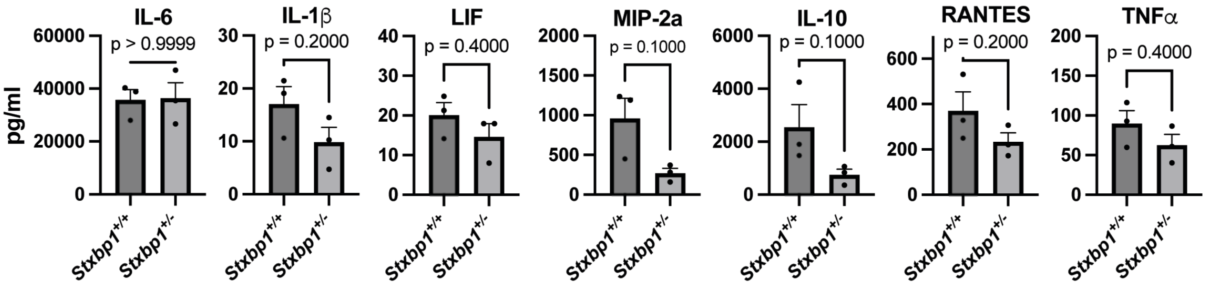

Supplementary Figure 3: Polymicrobial sepsis was induced by puncture of the cecum in ***Stxbp***$^{+/+}$ and ***Stxbp***$^{+/-}$ mice. The release of IL-6, IL-1β, LIF, MIP-2a, IL-10, RANTES, and TNFα was quantified 16 h after CLP operation. P-values determined by ordinary one-way ANOVA with Tukey's multiple comparisons test (SEM; *n=3, mice per group*).

Supplemental Figure 4

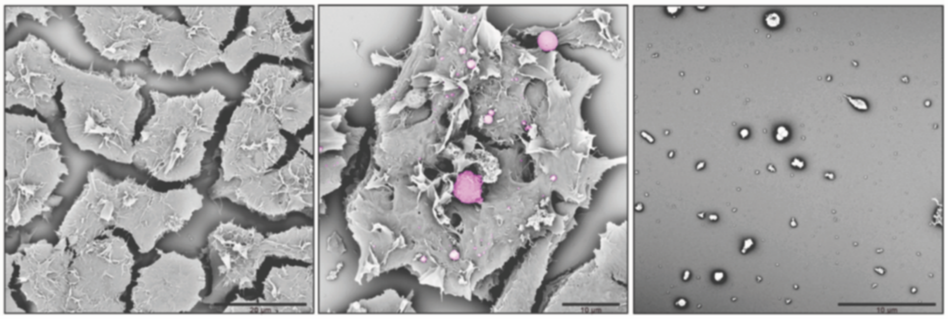

Supplementary Figure 4: Representative SEM pictures of HEK293 cells *(left panel)*, EVs attaching *(marked in magenta pseudo colors)* to HEK293 cells *(middle panel)*, and isolated EVs are shown *(right panel)*. Scale bar corresponds to 20 µm *(left panel)* and 10 µm *(middle)* and *(right panel)*. *(n=2 of different measurements)*.

Supplemental Figure 5

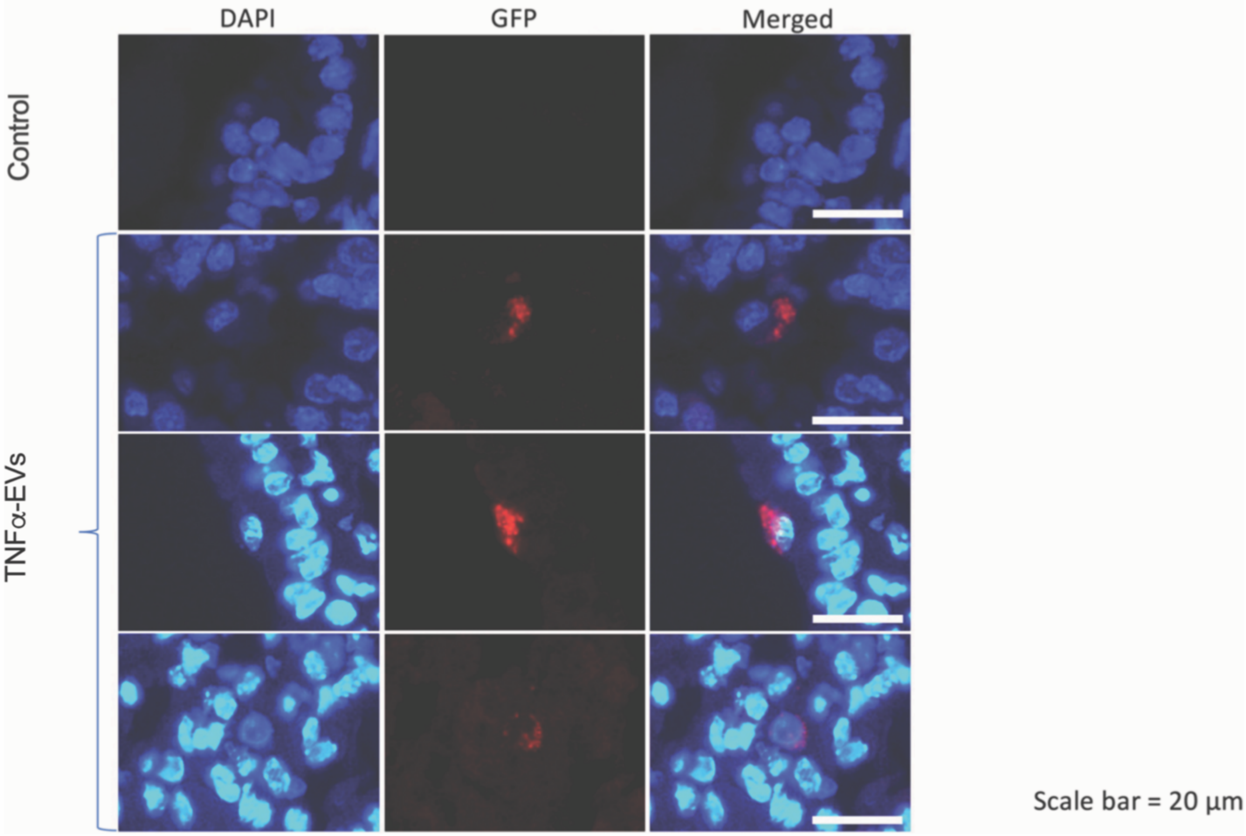

Supplementary Figure 5: Immunofluorescence images of figure 6. Mice were challenged with TNFR1-GFP EVs isolated from TNFR1 GFP stable cells that were stimulated with TNFα overnight *(EVs from 200 million cells/mouse)*. Thirty minutes after EV challenge lung samples were collected and analyzed by immunostaining. The nuclei of the cells in the lung tissue of a non-challenged mouse *(left panel)* and lung tissue from a EV challenged mouse *(middle panel)* were counterstained with DAPI *(blue)* and EVs were visualized with anti GFP AF594 *(red)*. Scale bar corresponds to 20 µm *(n=3 of different measurements)*.

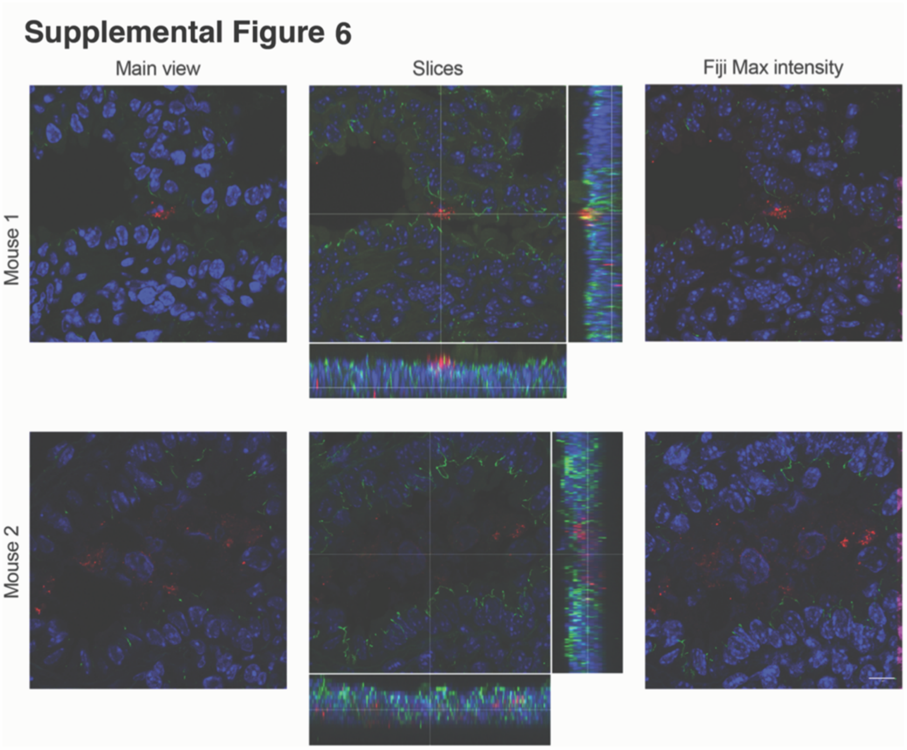

Supplementary Figure 6: Confocal images of figure 6. Confocal image *(right panel)* with EVs *(red)* and plasma membrane marker ZO-1 *(green) (*epithelial and endothelial cells*)*. The nuclei are counterstained with DAPI *(blue)*. Scale bar corresponds to 10 µm *(n=3 of different measurements)*.

Supplementary Figure 7

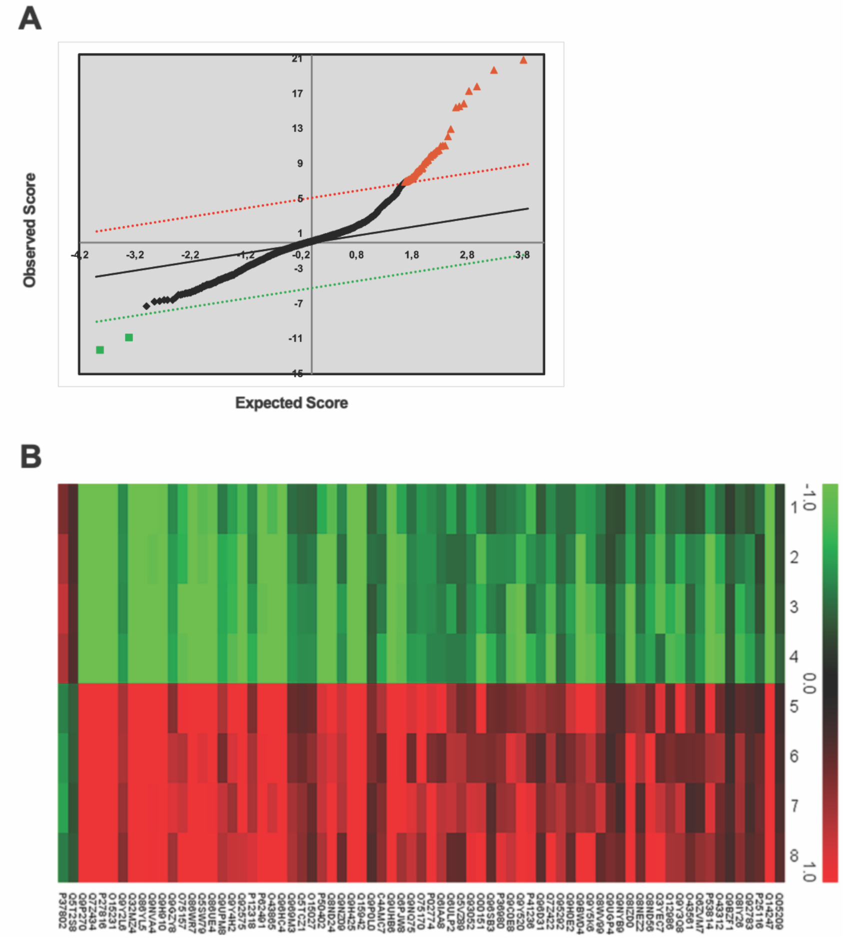

Supplementary Figure 7: A. Significance analysis of microarrays *(SAM)*. Significance analysis of microarrays analysis was performed to compare proteome sample isolates collected from polytrauma and sepsis patients. Over-expressed proteins are illustrated in red, down-regulated proteins are depicted in green. In total 68 proteins were found over-expressed, and 2 proteins were down-regulated *(q-value 0%, fold-change > 4.0)*. **B.** Heatmap. *Red* indicates highly expressed proteins and *Green* indicates lower expressed proteins with 4-fold change. Data points 1-4 are values from EVs isolated from polytrauma patients and 5-8 from EVs isolated from sepsis patients.
