# Supplementary material for: The role of extracellular vesicle fusion with target cells in triggering systemic inflammation
Source: Nat Commun. 2024 Feb 7;15:1150. doi: 10.1038/s41467-024-45125-1 (PMC10850166; doi:10.1038/s41467-024-45125-1)
Supplement: Supplementary file 3 — Reporting Summary [file 41467_2024_45125_MOESM3_ESM.pdf]

nature portfolio

Corresponding author(s): Heiko Herwald

Last updated by author(s): Dec 23, 2023

Reporting Summary

Nature Portfolio wishes to improve the reproducibility of the work that we publish. This form provides structure for consistency and transparency in reporting. For further information on Nature Portfolio policies, see our Editorial Policies and the Editorial Policy Checklist.

Statistics

For all statistical analyses, confirm that the following items are present in the figure legend, table legend, main text, or Methods section.

n/a	Confirmed	
☐	☒	The exact sample size (*n*) for each experimental group/condition, given as a discrete number and unit of measurement
☐	☒	A statement on whether measurements were taken from distinct samples or whether the same sample was measured repeatedly
☐	☒	The statistical test(s) used AND whether they are one- or two-sided<br>*Only common tests should be described solely by name; describe more complex techniques in the Methods section.*
☒	☐	A description of all covariates tested
☐	☒	A description of any assumptions or corrections, such as tests of normality and adjustment for multiple comparisons
☐	☒	A full description of the statistical parameters including central tendency (e.g. means) or other basic estimates (e.g. regression coefficient) AND variation (e.g. standard deviation) or associated estimates of uncertainty (e.g. confidence intervals)
☐	☒	For null hypothesis testing, the test statistic (e.g. *F*, *t*, *r*) with confidence intervals, effect sizes, degrees of freedom and *P* value noted<br>*Give P values as exact values whenever suitable.*
☒	☐	For Bayesian analysis, information on the choice of priors and Markov chain Monte Carlo settings
☒	☐	For hierarchical and complex designs, identification of the appropriate level for tests and full reporting of outcomes
☒	☐	Estimates of effect sizes (e.g. Cohen's *d*, Pearson's *r*), indicating how they were calculated

Our web collection on statistics for biologists contains articles on many of the points above.

Software and code

Policy information about availability of computer code

Data collection	Gel electrophoresis, ChemiDoc'" Imaging System (Bio-Rad).<br>Absorbance at different wavelength, Wal lac 1420 Workstation Sofrware version 3.00.<br>Flow cytometry using a Beckman Coulter Cytoflex (Beckman).<br>qPCR, The Applied Biosystems™ TaqMan™ Pre-Developed Assay Reagents from Thermo Fisher.<br>SEM images, PHENOM PROX scanning electron microscope.<br>Confocal images were acquired and processed with NIS-elements, version: 4.60.02, (Laboratory Imaging, Nikon, Tokyo, Japan).
Data analysis	GraphPad Prism 9.0 (GraphPad Software, San Diego, CA).<br>MS raw data files for the TMT set were merged for relative quantification and identification using Proteome Discoverer version 1.4 (Thermo Fisher Scientific). A database search for each set was performed with the Mascot search engine (Matrix Science) using the Homo Sapiens Swissprot database, version Mars 2017 with 553941sequences. MS peptide tolerance of 5 ppm and MS/MS tolerance for identification of 600 millimass units (mmu), tryptic peptides with zero missed cleavage and variable modifications of methionine oxidation, fixed modifications of cysteine alkylation, N-terminal TMT-label and lysine TMT-label were selected. The detected peptide threshold in the software was set to a significance of FDR 1% by searching against a reversed database and identified proteins were grouped by sharing the same sequences to minimize redundancy. For TMT quantification, the ratios of the TMT reporter ion intensities in HCD MS/MS spectra (m/z 126-131) from raw data sets were used. Ratios were derived by Proteome Discoverer using the following criteria: fragment ion tolerance as 3 mmu for the centroid peak with smallest delta mass and minimum intensity of 2000. Only peptides unique for a given protein were considered for relative quantitation, excluding those common to other isoforms or proteins of the same family. The quantification was normalized using the protein median. Calculations of the ratios were made by using a reference samples made from a mix of 4 of the samples or the control sample as

denominator.
Gene ontology (GO) enrichment analysis was performed using the Enrichr, a web-based tool providing various types of visualization summaries of collective functions of protein lists.

For manuscripts utilizing custom algorithms or software that are central to the research but not yet described in published literature, software must be made available to editors and reviewers. We strongly encourage code deposition in a community repository (e.g. GitHub). See the Nature Portfolio guidelines for submitting code & software for further information.

Data

Policy information about availability of data

All manuscripts must include a data availability statement. This statement should provide the following information, where applicable:

- Accession codes, unique identifiers, or web links for publicly available datasets
- A description of any restrictions on data availability
- For clinical datasets or third party data, please ensure that the statement adheres to our policy

The experimental data that support the findings of this work are available as Source Data.

All proteomics data in the study have been made public. The mass spectrometry proteomics data have been deposited to the ProteinXchange Consortium via the PRIDE partner respository, ...

Research involving human participants, their data, or biological material

Policy information about studies with human participants or human data. See also policy information about sex, gender (identity/presentation), and sexual orientation and race, ethnicity and racism.

Reporting on sex and gender	The study design did not specifically consider sex and gender as a factor. No separate data analysis for males and females was performed.
Reporting on race, ethnicity, or other socially relevant groupings	NA
Population characteristics	NA
Recruitment	NA
Ethics oversight	Informed consent was obtained from the patients, or the guardians included in this study. Samples from Helsingborg Hospital ICU were approved by the Ethics committee in Lund Dnr. 2015/467 and 2019/04558 and samples from Charles-University Prague were approved by the Ethics Committee of the Military University Hospital Prague Dnr. 108/9-36/2016-UVN.

Note that full information on the approval of the study protocol must also be provided in the manuscript.

Field-specific reporting

Please select the one below that is the best fit for your research. If you are not sure, read the appropriate sections before making your selection.

☒ Life sciences ☐ Behavioural & social sciences ☐ Ecological, evolutionary & environmental sciences

For a reference copy of the document with all sections, see nature.com/documents/nr-reporting-summary-flat.pdf

Life sciences study design

All studies must disclose on these points even when the disclosure is negative.

Sample size	No statistical methods were used to predetermine sample size estimates. Sample size was determined based on standards in the field and experiments to obtain statistical significance and reproducibility. At least triplicates were used to meet the minimal requirements for statistical analysis and the detailed sample size was demonstrated in the figure legends.
Data exclusions	No data was excluded from analysis.
Replication	To ensure the replication of the findings, experiments were repeated at different times as indicated in the figure legends. All experimental data was reliably reproduced in multiple independent experiments. For in vivo experiments, multiple mouse experiments were performed to ensure reproducibility, the exact number of animals was shown in the figure legends or as dots in the graphs.
Randomization	Animals were randomized according to the cage housing the animals.
Blinding	The experiments were performed with single-blindly.

Reporting for specific materials, systems and methods

We require information from authors about some types of materials, experimental systems and methods used in many studies. Here, indicate whether each material, system or method listed is relevant to your study. If you are not sure if a list item applies to your research, read the appropriate section before selecting a response.

Materials & experimental systems

n/a	Involved in the study
☐	☒ Antibodies
☐	☒ Eukaryotic cell lines
☒	☐ Palaeontology and archaeology
☐	☒ Animals and other organisms
☒	☐ Clinical data
☒	☐ Dual use research of concern
☒	☐ Plants

Methods

n/a	Involved in the study
☒	☐ ChIP-seq
☐	☒ Flow cytometry
☒	☐ MRI-based neuroimaging

Antibodies

Antibodies used

The follwing antibodies were used in the study:
CD66b-FITC (Cat# 305104; Clone: G10FS, BD Bioscience)
CD42b-PE-Cy5 (Cat# 551141; Clone: HI Pl, BD Bioscience)
CD14-BV421 (Cat# 301830; Clone: HCD14, Biolegend)
anti-lL1R1 antibody {Cat# sc-393998; Santa Cruz)
anti-plRAK4 (Cat#11927; Cell signaling Technology)
anti-MyD88 {Cat#4283; Cell signaling Technology)
anti-plRAK4, {Cat#11927; Cell signaling Technology)
anti-pTRAF2 antibodies {Cat#13908; Cell signaling Technology)
anti-TRADD antibodies {Cat# sc-46653; Santa Cruz)
anti-Munc 18-1 or STXBPl {Cat#ab3451; Abcam)
horseradish peroxidase-conjugated goat anti-rabbit lgG {Cat#172-1019; Bio-Rad)
anti-GAPDH {10494-1-AP; Proteintech)

Validation

All antibodies were validated by vendors:

CD66b-FITC; https://www.citeab.com/antibodies/521451-305104-fitc-anti-human-cd66b-g10f5-monoclonal?des=b8239f6ace2d47f0
CD42b-PE-Cy5; https://www.citeab.com/antibodies/2412461-551141-bd-pharmingen-pe-cy-5-mouse-anti-human-cd42b?des=334b657cd46f3e78
CD14-BV421: https://www.biolegend.com/ja-jp/products/brilliant-violet-421-anti-human-cd14-antibody-7321?GroupID=BLG4926
anti-lL1R1 antibody; https://www.scbt.com/p/il-1ri-antibody-h-8
anti-plRAK4; https://www.cellsignal.com/products/primary-antibodies/phospho-irak4-thr345-ser346-d6d7-rabbit-mab/11927
anti-MyD88; https://www.cellsignal.com/products/primary-antibodies/myd88-d80f5-rabbit-mab/4283
anti-plRAK4; https://www.cellsignal.com/products/primary-antibodies/phospho-irak4-thr345-ser346-d6d7-rabbit-mab/11927
anti-pTRAF2; https://www.cellsignal.com/products/primary-antibodies/phospho-traf2-ser11-e2b6l-rabbit-mab/13908
anti- TRADD; https://www.scbt.com/p/tradd-antibody-a-5?requestFrom=search
anti-Munc 18-1; https://www.abcam.com/en-se/products/primary-antibodies/munc18-1-antibody-ab3451
anti-GAPDH; https://www.ptglab.com/products/GAPDH-Antibody-10494-1-AP.htm

Eukaryotic cell lines

Policy information about cell lines and Sex and Gender in Research

Cell line source(s)

HEK293 (ATCC® CRL-1573™) was purchased from American Type Culture Collection.
RAW-Blue™, THP1-Dual™, THP-1-Dual™ KO-MyD88, HEK-Dual™ TNFa, HEK-Blue™ IL-1b, and HEK-Blue™ IL-10 reporter cells (hkb-TNFa, hkb-il1r, and hkb-il10) were obtained from InvivoGen, San Diego, CA, USA.

Authentication

All cell lines are authenticated by Invivogen and ATCC. No further validation was performed.

Mycoplasma contamination

All cell lines were tested negative for mycoplasma contamination by Invivogen and ATCC.

Commonly misidentified lines
(See ICLAC register)

We did not use a commonly misidentified cell line in this study.

Animals and other research organisms

Policy information about studies involving animals; ARRIVE guidelines recommended for reporting animal research, and Sex and Gender in Research

Laboratory animals	BALB/c and C57Bl6 {Stxbpl +/+ & Stxbpl +/- mice) (female, 6-9 weeks).
Wild animals	No wild animal were involved in this study.
Reporting on sex	Gender was not considered in the data analysis.
Field-collected samples	Not involved.
Ethics oversight	Animal experiments were performed according to protocols approved by the Local Ethics Committee at Lund University (Malmö-Lundsdjurförsöksetiskanämnd: Dnr 5.8.18-18-01753/2022).

Note that full information on the approval of the study protocol must also be provided in the manuscript.

Flow Cytometry

Plots

Confirm that:

- [x] The axis labels state the marker and fluorochrome used (e.g. CD4-FITC).
- [x] The axis scales are clearly visible. Include numbers along axes only for bottom left plot of group (a 'group' is an analysis of identical markers).
- [x] All plots are contour plots with outliers or pseudocolor plots.
- [x] A numerical value for number of cells or percentage (with statistics) is provided.

Methodology

Sample preparation	CD42b receptor transfer to neutrophils and monocytes cell surface was investigated by flow cytometry using a Beckman Coulter Cytoflex (Beckman). Citrated blood from five healthy donors was incubated with 0.5 µg/ml E. coli O111:B4 LPS (Sigma-Aldrich) for 15 min at 37 °C. 100 µl of blood was used and red blood cells were lysed with a BD Phosflow Lyse/Fix 5X. After lysis, samples were washed once with 0.5% BSA in PBS and incubated with CD66b-FITC (Clone: G10F5, BD Bioscience), CD42b-PE-Cy5 (Clone: HIP1, BD Bioscience) and CD14-BV421 (Clone: HCD14, BioLegend) antibodies were added (1:50) to the cell suspension and incubated for 60 min at 37 °C in the dark. For one donor, the manual gating analysis using FMO, and gating controls was carried out. Samples were washed once with 0.5% BSA in PBS and the cell pellet was resuspended in 300 µl of washing buffer. The percentage of CD42b on the cell surface was calibrated with control cells that were not treated with LPS. The results are presented as mean values ± s.e.m.
Instrument	Beckman Coulter Cytoflex (Beckman)
Software	CytExpert
Cell population abundance	Cell sorting was not employed
Gating strategy	For one donor, using the FSC/SSC gating, debris was removed by gating on the main cell population. The manual gating analysis using FMO, and gating controls was carried out.

- [ ] Tick this box to confirm that a figure exemplifying the gating strategy is provided in the Supplementary Information.
